# Integrating Prostate‐Specific Antigen Density and Prostate Imaging Reporting and Data System Scores to Optimize Detection of Clinically Significant Prostate Cancer: A Multivariable Risk Model Approach

**DOI:** 10.1002/jcla.70218

**Published:** 2026-04-04

**Authors:** Yunus Kayali

**Affiliations:** ^1^ Department of Urology University of Health Sciences, Kartal Dr. Lutfi Kirdar City Hospital Istanbul Turkey

**Keywords:** decision support techniques, logistic models, multiparametric magnetic resonance imaging, predictive value of tests, prostate‐specific antigen, prostatic neoplasms, receiver operating characteristic curve, risk assessment

## Abstract

**Background:**

Prebiopsy multiparametric MRI (mpMRI) reported using the Prostate Imaging Reporting and Data System (PI‐RADS) improves prostate cancer triage, yet false‐positive findings remain common and may drive unnecessary biopsy. Prostate‐specific antigen density (PSAD) is an inexpensive laboratory‐derived adjunct that may refine MRI‐based risk stratification.

**Methods:**

We retrospectively screened 713 transrectal ultrasound (TRUS)‐guided biopsy episodes from a single experienced urologist's practice at a tertiary referral hospital between October 2022 and August 2025 and included 375 men with prebiopsy mpMRI reported using PI‐RADS version 2.1 and complete data for prespecified predictors. All patients underwent systematic 12‐core TRUS‐guided biopsy. Clinically significant prostate cancer (csPCa) was defined as International Society of Urological Pathology (ISUP) grade group ≥ 2. We developed logistic regression models combining PSAD and PI‐RADS (parsimonious model) and adding age and digital rectal examination (DRE) (full model). Discrimination (AUC), calibration, and clinical utility (decision curve analysis) were assessed, and the incremental value of PSAD beyond PI‐RADS for csPCa was quantified using IDI and continuous NRI.

**Results:**

csPCa was present in 93/375 (24.8%) and any prostate cancer in 144/375 (38.4%). For csPCa prediction, AUC was 0.746 for PI‐RADS and 0.760 for PSAD; the combined PSAD+PI‐RADS model achieved AUC 0.798 and the full model AUC 0.794. Adding PSAD to PI‐RADS improved IDI (0.0528; *p* < 0.001) and total continuous NRI (0.3926; *p* < 0.001), driven mainly by improved down‐classification of non‐csPCa cases.

**Conclusions:**

Integrating PSAD with PI‐RADS improved csPCa risk stratification compared with PI‐RADS alone, with predominant benefit as a biopsy‐sparing, rule‐out adjunct. External validation is required for clinical implementation.

## Graphical abstract

For the detection of clinically significant prostate cancer, incorporating PSA density into PI‐RADS‐based assessment improved clinical net benefit compared with PI‐RADS alone. The addition of DRE and age to this combined model produced only marginal further gain, indicating that most of the incremental clinical value was derived from PSA density rather than from increased model complexity.

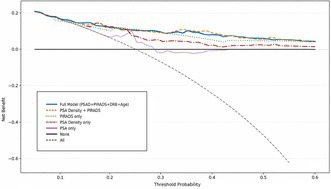

## 1 Introduction

Prostate cancer (PCa) remains one of the most frequently diagnosed malignancies in men, and contemporary diagnostic pathways must balance early detection of clinically significant prostate cancer (csPCa) against harms of unnecessary biopsy and overdiagnosis of indolent disease [1]. Serum prostate‐specific antigen (PSA) testing is widely used for risk stratification, yet its limited specificity motivates adjunctive approaches that improve selection for biopsy, particularly in diagnostically challenging ranges.

Prebiopsy mpMRI has transformed diagnostic work‐up by localizing suspicious lesions and standardizing reporting via PI‐RADS [2]. Prospective studies, including PROMIS and PRECISION, support mpMRI‐guided diagnostic pathways by improving detection of csPCa while reducing unnecessary biopsies [3, 4]. However, PI‐RADS performance varies across centers, with a low and heterogeneous positive predictive value, implying variable false‐positive rates and potentially unnecessary biopsies [5].

Prostate‐specific antigen density (PSAD), defined as serum PSA divided by prostate volume, is a pragmatic laboratory‐derived metric that contextualizes PSA for gland size. Risk‐adapted early detection strategies increasingly incorporate PSAD alongside MRI to support biopsy decisions [6, 7, 8]. Prior studies indicate that PSAD provides incremental, complementary value to PI‐RADS for improving risk stratification, particularly for equivocal PI‐RADS 3 lesions, and may support biopsy avoidance strategies without materially compromising detection of clinically significant disease [9, 10, 11, 12].

Accordingly, we developed and internally evaluated an interpretable multivariable logistic regression model integrating PSAD and PI‐RADS, with additional readily available clinical variables, to optimize csPCa detection among men undergoing TRUS‐guided biopsy. Specifically, we sought to quantify the discriminatory performance of PSAD–PI‐RADS integration, to evaluate its potential clinical utility in guiding biopsy decisions using decision curve analysis, and to determine the incremental predictive value beyond individual predictors using contemporary reclassification metrics. Collectively, this work aims to provide a pragmatic, internally validated approach for incorporating PSAD alongside PI‐RADS in csPCa risk assessment.

## 2 Materials and Methods

### 2.1 Study Design, Setting, and Data Collection

This retrospective, single‐center cohort study was conducted at a tertiary referral hospital. Because digital rectal examination (DRE) is clinician dependent and may vary substantially between examiners, the cohort was restricted to biopsy episodes managed by a single experienced urologist to standardize DRE assessment and clinical documentation across participants. The study protocol was approved by the institutional ethics committee (Ref. no: 2025/010.99/20/22).

We screened 713 consecutive TRUS‐guided biopsy episodes assessed by the same urologist between October 2022 and August 2025. Eligibility required prebiopsy mpMRI with an available PI‐RADS report and complete data for prespecified predictors (PSA, prostate volume, PI‐RADS category, DRE findings, and age). We restricted the cohort to men with serum PSA concentrations within the diagnostically challenging range of 2.5–20 ng/mL. Exclusion criteria were applied sequentially, and each excluded case was assigned to the first applicable criterion: no mpMRI performed or no PI‐RADS report (*n* = 63); technically inadequate biopsy (fewer than 8 cores) or missing pathology report (*n* = 39); prior PCa diagnosis or prior prostate volume‐altering surgery (e.g., TURP or HoLEP) (*n* = 47); current 5‐alpha‐reductase inhibitor use (finasteride or dutasteride) (*n* = 134); PSA outside 2.5–20 ng/mL (*n* = 16); and factors likely to transiently alter PSA within the preceding 4–6 weeks (prostate manipulation, acute infection/inflammation, or acute urinary retention) (*n* = 39). After exclusions (*n* = 338), the final analytic cohort comprised 375 men. No patients were excluded on the basis of biopsy outcome; therefore, both cancer and non‐cancer cases were included, enabling assessment of discrimination between csPCa and benign or indolent findings.

### 2.2 MRI Acquisition and Reporting

Prostate MRI was performed using the institutional mpMRI protocol consistent with PI‐RADS version 2.1 recommendations, including high‐resolution T2‐weighted imaging and diffusion‐weighted imaging with apparent diffusion coefficient maps; dynamic contrast‐enhanced imaging was acquired per protocol when indicated. Examinations were interpreted by radiologists using PI‐RADS version 2.1 [2]. The highest PI‐RADS category reported for the examination was used for analysis.

### 2.3 Biopsy Procedure and Histopathology

All included patients underwent TRUS‐guided transrectal biopsy using a systematic sampling strategy. Sampling consisted of a standard 12 core scheme (bilateral apex, mid‐gland, and base, including parasagittal and lateral regions). Histopathology was reported by dedicated genitourinary pathologists. Cancer grade was assigned according to ISUP grade groups. Any PCa was defined as ISUP grade group ≥ 1, and csPCa as ISUP grade group ≥ 2.

### 2.4 Predictors and Outcomes

Candidate predictors were selected a priori based on clinical availability and prior literature: age (years), DRE result (positive/suspicious vs. negative), PI‐RADS category (ordinal), and PSAD (ng/mL/mL). DRE was recorded as positive/suspicious when findings such as induration, asymmetry, or a palpable nodule were documented. PSAD was calculated as serum PSA divided by prostate volume as recorded in the clinical record. PSA was evaluated as a single predictor but was not included in multivariable models because PSAD already incorporates PSA, reducing redundancy and collinearity.

### 2.5 Statistical Analysis

We developed two logistic regression models: (i) a parsimonious model combining PSAD and PI‐RADS and (ii) a full model adding DRE and age. Model performance assessment followed established frameworks emphasizing discrimination, calibration, and clinical utility [13, 14]. Discrimination was quantified by the area under the receiver operating characteristic curve (AUC) with 95% confidence intervals. Calibration was assessed visually using a calibration plot comparing observed and predicted csPCa risk across strata of predicted probability. Clinical utility was evaluated using decision curve analysis across threshold probabilities relevant to biopsy decision‐making [15]. Incremental value of adding PSAD to PI‐RADS for csPCa prediction was quantified using the integrated discrimination improvement (IDI) and the continuous (category‐free) net reclassification improvement (cNRI), with separate components for events (csPCa) and nonevents (non‐csPCa) [16]. As a sensitivity analysis to evaluate model robustness and mitigate potential overfitting, we fitted a ridge‐penalized logistic regression using an 80/20 stratified train–test split (random_state = 42) and reported standardized coefficients together with test‐set performance metrics (AUC, log‐loss, and Brier score).

## 3 Results

Between October 2022 and August 2025, 713 TRUS‐guided biopsy episodes from a single urologist's practice—selected to standardize DRE assessment—were screened; after applying prespecified exclusion criteria, 375 men were included in the analytic cohort. Baseline characteristics are summarized in Table 1.

**TABLE 1:** Characteristics of the study cohort (*n* = 375).

Characteristic	Value
Total patients (*n*)	375
Age, years (median [IQR])	62 [56–68]
Serum PSA, ng/mL (median [IQR])	6.10 [4.30–8.67]
PSAD, ng/mL/mL (median [IQR])	0.100 [0.074–0.165]
Outcome status, *n* (%)	
csPCa (ISUP GG ≥ 2)	93 (24.8%)
non‐csPCa (benign + ISUP GG1)	282 (75.2%)
DRE result, *n* (%)	
Positive	57 (15.2%)
Negative	318 (84.8%)
PI‐RADS distribution, *n* (%)	
PI‐RADS 2	150 (40.0%)
PI‐RADS 3	96 (25.6%)
PI‐RADS 4	105 (28.0%)
PI‐RADS 5	24 (6.4%)

*Note:* No PI‐RADS 1 lesions were observed.

Abbreviations: csPCa, clinically significant prostate cancer; DRE, digital rectal examination; ISUP GG, International Society of Urological Pathology Grade Group; PI‐RADS, Prostate Imaging Reporting and Data System; PSA, prostate‐specific antigen; PSAD, prostate‐specific antigen density.

On systematic biopsy, any PCa was detected in 144/375 (38.4%). Clinically significant disease (ISUP grade group ≥ 2) was present in 93/375 (24.8%), while 51/375 (13.6%) had ISUP grade group 1 and 231/375 (61.6%) had benign histopathology.

**FIGURE 1:**
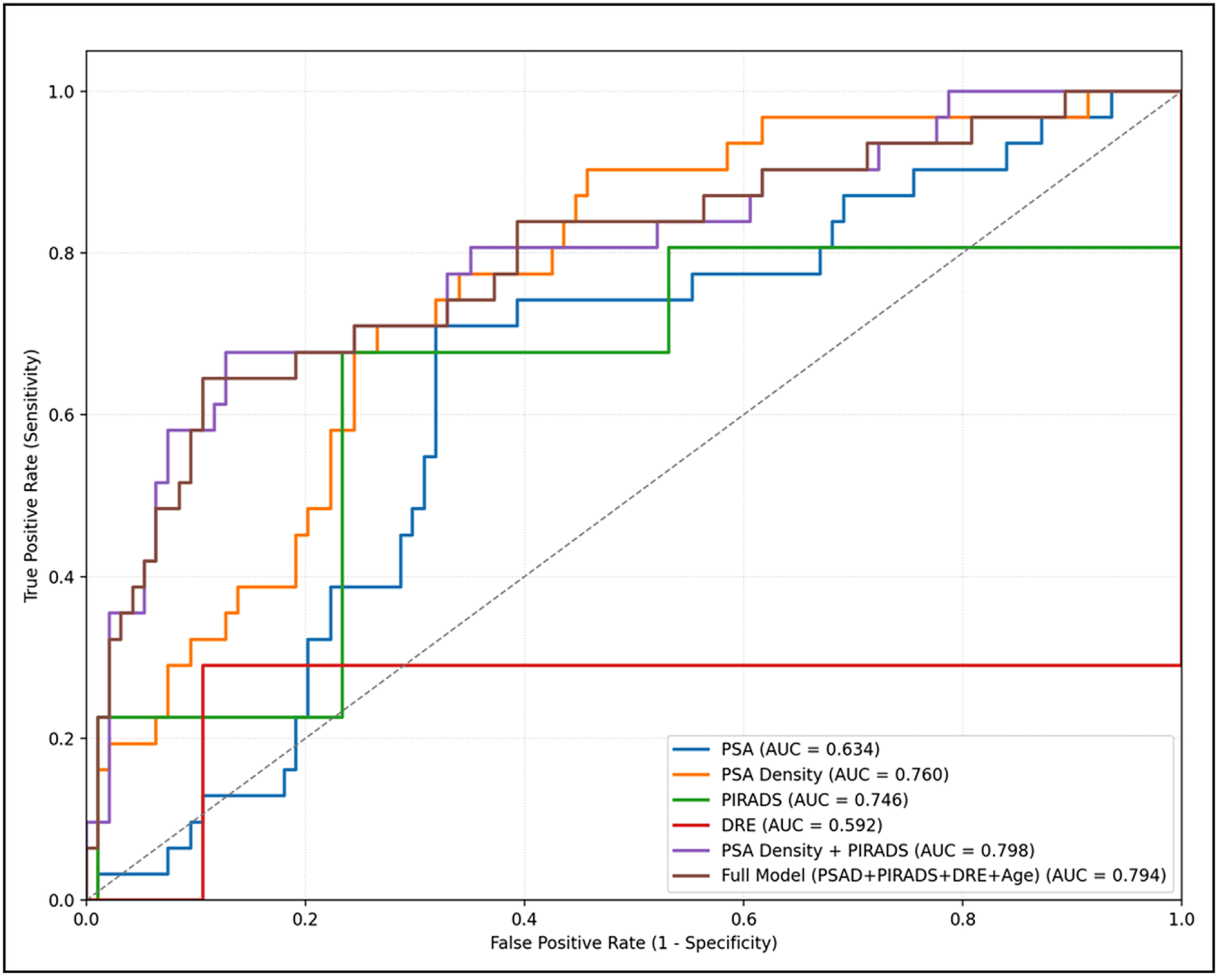
Receiver operating characteristic curves for the detection of clinically significant prostate cancer. Receiver operating characteristic (ROC) curves comparing the discriminative performance of PSA, PSA density, PI‐RADS, DRE, PSA density plus PI‐RADS, and the full multivariable model (PSA density + PI‐RADS + DRE + age) for the detection of clinically significant prostate cancer (csPCa). The area under the curve (AUC) for each model is shown in the figure. The diagonal dashed line represents no discrimination. AUC, area under the curve; csPCa, clinically significant prostate cancer; DRE, digital rectal examination; PI‐RADS, Prostate Imaging Reporting and Data System; PSA, prostate‐specific antigen.

**FIGURE 2:**
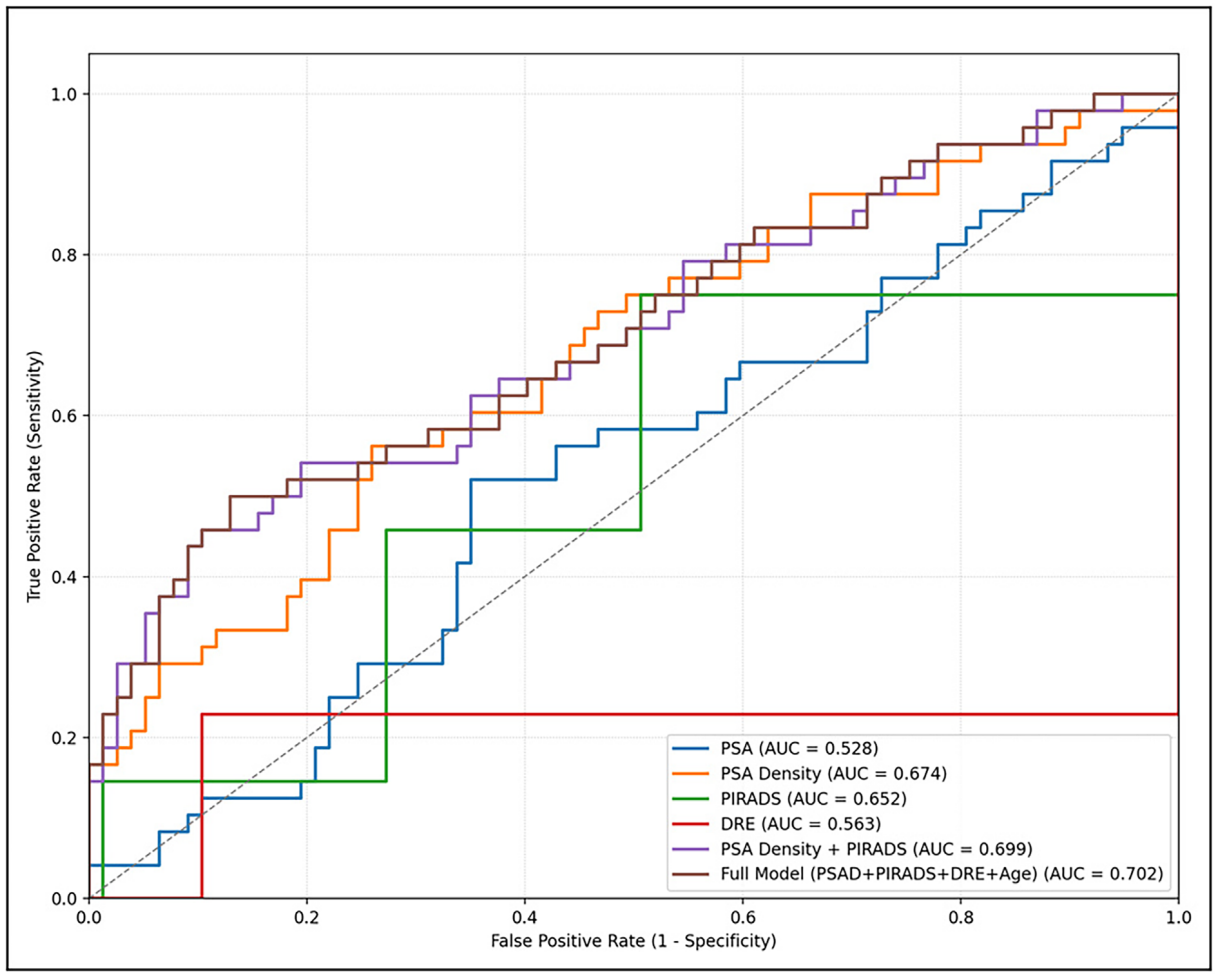
Receiver operating characteristic curves for the detection of prostate cancer. Receiver operating characteristic (ROC) curves comparing the performance of PSA, PSA density, PI‐RADS, DRE, PSA density plus PI‐RADS, and the full multivariable model (PSA density + PI‐RADS + DRE + age) for the detection of overall prostate cancer (PCa). The area under the curve (AUC) for each model is displayed in the figure. The diagonal dashed line indicates the line of no discrimination. AUC, area under the curve; DRE, digital rectal examination; PCa, prostate cancer; PI‐RADS, Prostate Imaging Reporting and Data System; PSA, prostate‐specific antigen.

Discrimination results are presented in Table 2. For csPCa prediction, AUC was 0.746 (95% CI, 0.693–0.798) for PI‐RADS and 0.760 (95% CI, 0.707–0.813) for PSAD. The combined PSAD+PI‐RADS model yielded AUC 0.798 (95% CI, 0.749–0.848), with similar performance for the full model adding DRE and age (AUC 0.794; 95% CI, 0.744–0.843) (Figure 1). For any PCa prediction, discrimination was more modest; the full model achieved AUC 0.702 (95% CI, 0.648–0.755) (Figure 2).

**TABLE 2:** Discrimination (AUC) for PCa and csPCa (primary endpoint).

Predictor/Model	PCa presence (AUC) (95% CI)	csPCa presence (AUC) (95% CI)
PSA	0.528 (0.472–0.591)	0.634 (0.576–0.698)
DRE	0.563 (0.525–0.603)	0.592 (0.540–0.640)
PSAD	0.674 (0.617–0.727)	0.760 (0.706–0.815)
PI‐RADS	0.652 (0.600–0.704)	0.746 (0.687–0.805)
PSAD + PI‐RADS	0.699 (0.646–0.757)	0.798 (0.742–0.859)
Full Model (PSAD + PI‐RADS + DRE + Age)	0.702 (0.649–0.763)	0.794 (0.737–0.855)

Abbreviations: AUC, area under the ROC curve; CI, confidence interval; DRE, digital rectal examination; PI‐RADS, Prostate Imaging Reporting and Data System; PSA, prostate‐specific antigen; PSAD, prostate‐specific antigen density.

In multivariable analysis (Table S1), PSAD and PI‐RADS were independently associated with csPCa. PSAD (per 0.1 ng/mL/mL increase) had an odds ratio (OR) of 1.63 (95% CI, 1.27–2.09; *p* < 0.001) and PI‐RADS (per 1‐point increase) an OR of 2.65 (95% CI, 1.94–3.62; *p* < 0.001). DRE and age were not statistically significant in the full model. Consistent with these findings, adding DRE and age did not materially improve discrimination beyond the PSAD+PI‐RADS model.

Incremental value of PSAD beyond PI‐RADS for csPCa is summarized in Table 3. Adding PSAD improved IDI by 0.0528 (95% CI, 0.025–0.080; *p* < 0.001) and yielded a significant total cNRI of 0.3926 (95% CI, 0.137–0.648; *p* = 0.003). Decomposition demonstrated that the net reclassification benefit was driven primarily by more accurate down‐classification of non‐csPCa cases (NRI_nonevent 0.4894; *p* = 0.001), with no significant improvement in up‐classification of csPCa events (NRI_event −0.0968; *p* = 0.392).

**TABLE 3:** Incremental value of PSAD + PI‐RADS compared with PI‐RADS alone for csPCa.

Metric	Value	95% CI	*p*
Integrated discrimination improvement (IDI)	0.0528	0.0182–0.1094	< 0.001
Continuous NRI (cNRI), total	0.3926	0.2361–0.7138	< 0.001
NRI_nonevent (non‐csPCa; rule‐out component)	+0.4894	0.4043–0.6099	< 0.001
NRI_event (csPCa; rule‐in component)	−0.0968	−0.2258–0.1613	0.717

*Note:* IDI and continuous NRI were computed from predicted probabilities; uncertainty was quantified using stratified bootstrap resampling (2000 iterations).

Abbreviations: cNRI, continuous (category‐free) NRI; csPCa, clinically significant prostate cancer; IDI, integrated discrimination improvement; NRI, net reclassification improvement.

**FIGURE 3:**
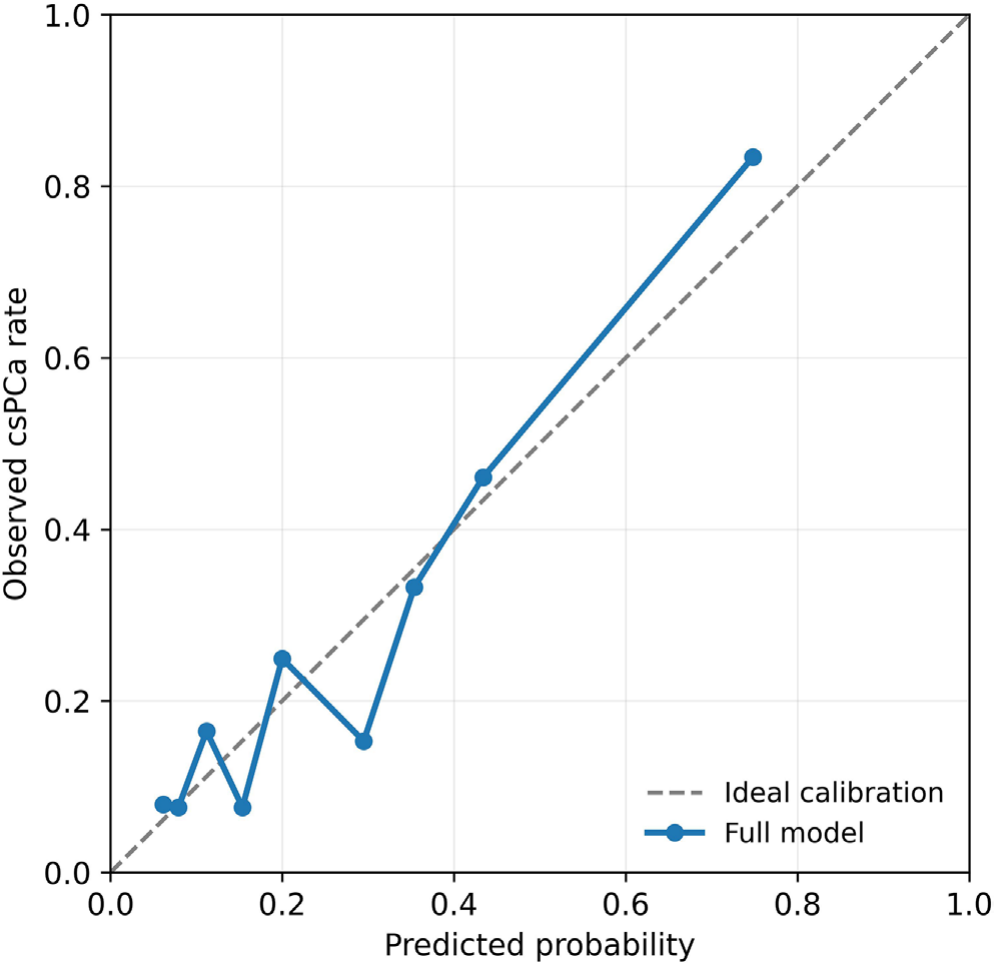
Calibration plot of the full multivariable model for clinically significant prostate cancer. Calibration plot of the full multivariable model (PSA density + PI‐RADS + DRE + age) for predicting clinically significant prostate cancer (csPCa). The x‐axis shows predicted probability and the y‐axis shows the observed csPCa rate across grouped predictions. The dashed diagonal line represents ideal calibration, whereas the solid line with markers represents the observed calibration of the model. csPCa, clinically significant prostate cancer; DRE, digital rectal examination; PI‐RADS, Prostate Imaging Reporting and Data System; PSA, prostate‐specific antigen.

**FIGURE 4:**
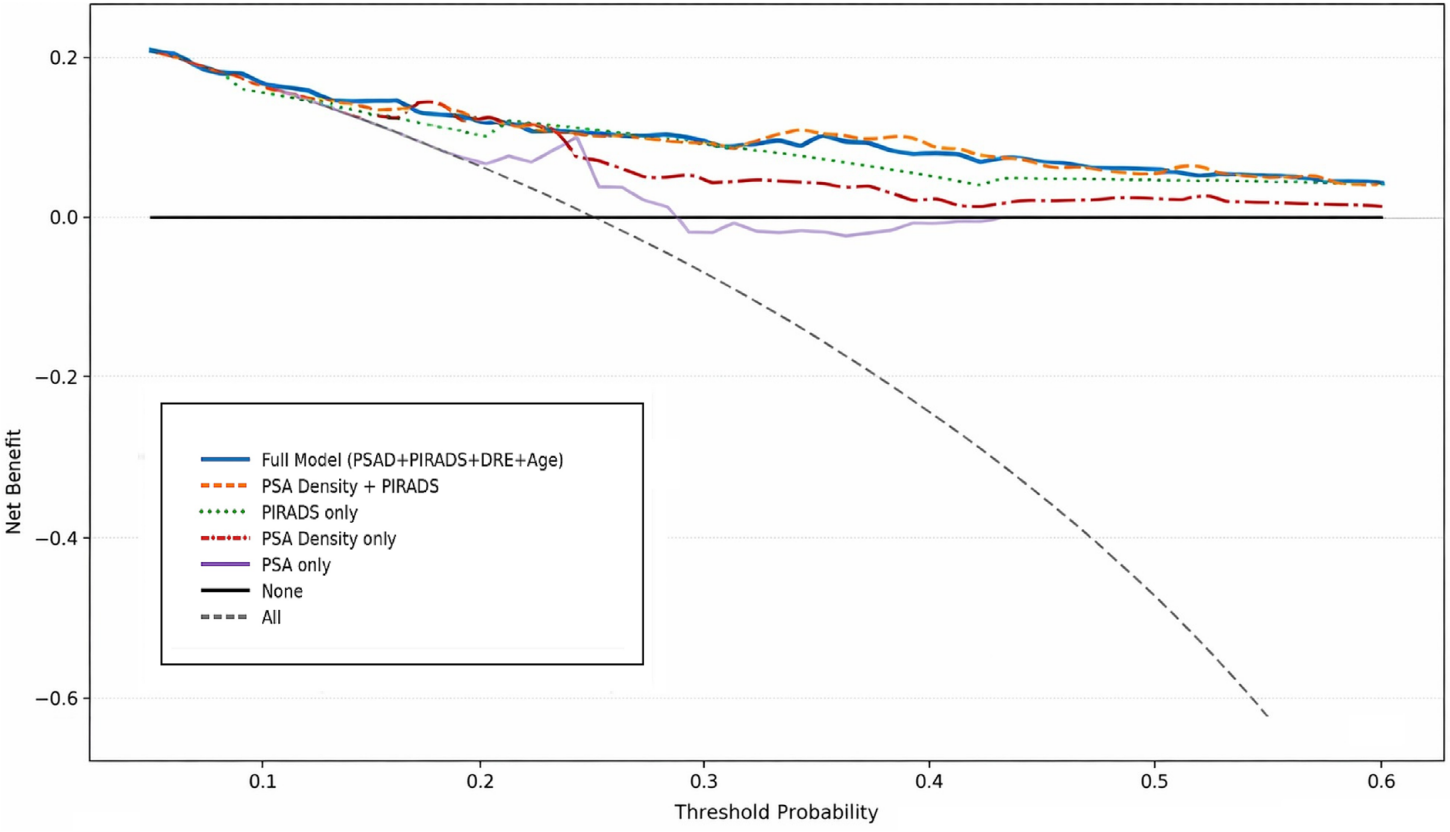
Decision curve analysis for clinically significant prostate cancer. Decision curve analysis showing the net benefit of PSA, PSA density, PI‐RADS alone, PSA density alone, PSA density plus PI‐RADS, and the full multivariable model (PSA density + PI‐RADS + DRE + age) for predicting clinically significant prostate cancer (csPCa) across a range of threshold probabilities. The horizontal solid line indicates the strategy of biopsying no patients, and the dashed line indicates the strategy of biopsying all patients. Higher net benefit indicates greater potential clinical utility. csPCa, clinically significant prostate cancer; DRE, digital rectal examination; PI‐RADS, Prostate Imaging Reporting and Data System; PSA, prostate‐specific antigen.

Calibration of the full model for csPCa is shown in Figure 3, and visual inspection suggested generally acceptable agreement between predicted and observed csPCa risk, with modest deviations in intermediate‐risk strata. Decision curve analysis (Figure 4) suggested higher net benefit for the PSAD + PI‐RADS and full models compared with single predictors and default strategies across clinically relevant threshold probabilities. On decision curve analysis, the PSAD+PI‐RADS and full models yielded higher net benefit than PI‐RADS alone and default strategies across most threshold probabilities shown (approximately 0.05–0.60).

In ridge‐penalized sensitivity analysis (Table S2), standardized coefficients were largest for PI‐RADS and PSAD, and test‐set performance remained comparable (AUC 0.799), supporting model stability.

## 4 Discussion

In this tertiary referral, single‐center cohort of men undergoing prebiopsy mpMRI and subsequent TRUS‐guided systematic biopsy, we developed and internally evaluated an interpretable logistic regression model integrating PSAD and PI‐RADS for prediction of csPCa. The combined PSAD+PI‐RADS model demonstrated strong discrimination (AUC 0.798) and performed similarly to a slightly expanded model incorporating DRE and age.

Our findings align with the evolving evidence base supporting PSAD as a valuable adjunct to MRI. Distler et al. showed that combining PSAD with PI‐RADS improves the negative predictive value of MRI assessment and may allow avoidance of approximately 20% of unnecessary biopsies by better ruling out clinically significant disease [9]. The complementary role of PSAD has also been reported in contemporary cohorts, including a large multi‐institutional collaborative focusing on PI‐RADS 3 lesions (Drevik et al.) and studies highlighting PSAD as complementary to PI‐RADS for csPCa risk stratification (Frisbie et al.) [10, 11]. In addition, combining PSA‐derived metrics with MRI may enhance biopsy avoidance strategies, consistent with reports using biparametric MRI and PSAD to help rule out higher‐grade disease in biopsy‐naïve men [12].

A clinically important observation concerns the pattern of incremental value. Although the improvement in AUC over PI‐RADS alone was numerically modest, IDI and total cNRI suggested meaningful gains in average risk separation and patient‐level reclassification. Notably, decomposition of cNRI indicated that the predominant benefit arose from more accurate down‐classification of men without csPCa, supporting the model's role as a biopsy‐sparing, rule‐out adjunct rather than a rule‐in tool. This interpretation is consistent with EAU risk‐adapted early detection recommendations that integrate mpMRI findings with PSA‐derived measures (including PSA density) to individualize biopsy decisions, and with contemporary evidence supporting PSAD as complementary to PI‐RADS for biopsy avoidance strategies [6, 7, 8].

The positive predictive value of PI‐RADS varies substantially across centers, underscoring heterogeneity in prostate MRI performance and interpretation [5]. PSAD offers an objective laboratory‐derived measure that can partially mitigate MRI false positives by contextualizing PSA relative to gland size. This practicality positions PSAD as a low‐cost, readily implementable bridge between laboratory testing and MRI reporting in multidisciplinary prostate cancer pathways.

Decision curve analysis demonstrated higher net benefit for models incorporating PSAD and PI‐RADS than for single predictors across clinically relevant threshold probabilities (Figure 4). Because biopsy thresholds depend on patient values, local practice, and downstream resource considerations, future studies should focus on clinically actionable risk cutoffs and on evaluating biopsy avoidance, missed csPCa rates, and downstream treatment outcomes under proposed decision rules.

Barone et al. evaluated the reliability of mpMRI in men undergoing repeat biopsy after a previous negative result and showed that, despite a lower overall prevalence of cancer in this setting, mpMRI retained clinically useful capability to identify csPCa, while also highlighting that false‐positive MRI findings remain a practical challenge in real‐world pathways [17]. In relation to our findings, this provides an important clinical context: in a systematic TRUS‐biopsy workflow, where MRI findings may otherwise drive biopsy decisions with variable specificity, adding PSAD to PI‐RADS yielded only a numerically modest change in AUC but a meaningful improvement in reclassification that was driven predominantly by down‐classification of men without csPCa (i.e., improved nonevent classification). Taken together, our results extend the evidence base by quantifying how a PSA‐derived, laboratory‐accessible metric can complement MRI‐based assessment specifically by mitigating unnecessary biopsy prompts arising from MRI false positives—an issue that becomes particularly consequential when pretest probability is lower, such as in repeat‐biopsy pathways emphasized by Barone et al. [17].

In a retrospective single‐center study of 630 biopsy‐naïve men undergoing prebiopsy mpMRI followed by systematic 12‐core TRUS biopsy, Massanova et al. reported that PI‐RADS and PSAD were independent predictors of both overall PCa and csPCa, and that combining these measures could help identify a subgroup at sufficiently low risk to consider deferring biopsy [18]. In our cohort—restricted to PSA concentrations within a diagnostically challenging range—PSAD likewise remained a significant contributor when considered alongside PI‐RADS, and the incremental value analysis suggested that the principal clinical gain was improved recognition of men unlikely to harbor csPCa. Taken together, these findings reinforce the concept that PSAD meaningfully complements MRI‐based stratification, with the greatest potential impact in biopsy avoidance among lower‐risk patients.

This study has several limitations. First, the retrospective, single‐center design may constrain external validity, and all reported performance estimates reflect internal evaluation. Second, although restricting the cohort to a single urologist enhanced standardization of DRE and reduced inter‐examiner variability, it may also introduce selection related to local referral patterns and clinician‐specific practice style. Third, eligibility criteria were intentionally focused on men with PSA concentrations within the diagnostically challenging range of 2.5–20 ng/mL, and patients receiving 5‐alpha‐reductase inhibitors were excluded; consequently, the findings may not extrapolate to populations outside this PSA range or to settings with different PSA distributions. Finally, systematic TRUS‐guided biopsy is an imperfect reference standard and may fail to detect some csPCa, which could bias effect estimates toward the null and attenuate apparent model performance. External validation in independent, multicenter cohorts with harmonized MRI reporting, biopsy protocols, and laboratory measurements would strengthen evidence for transportability and support broader implementation.

Overall, integrating prostate‐specific antigen density with PI‐RADS provided a measurable improvement in risk stratification for clinically significant prostate cancer beyond PI‐RADS alone in this cohort. Although gains in overall discrimination were numerically modest, reclassification analyses suggested that adding PSAD meaningfully refined patient‐level risk estimates, with the predominant incremental value arising from more accurate identification and down‐classification of men unlikely to harbor csPCa. In practical terms, these findings support PSAD as a readily obtainable, laboratory‐derived complement to MRI interpretation that may help temper false‐positive–driven biopsy decisions and improve selection of men who can reasonably defer biopsy within MRI‐informed pathways. At the same time, given the single‐center design and internal evaluation, the present results should be interpreted as supportive evidence for a pragmatic, biopsy‐sparing rule‐out adjunct rather than definitive proof of a universally generalizable decision rule.

## Funding

This research received no specific grant from any funding agency in the public, commercial, or not‐for‐profit sectors.

## Ethics Statement

This study was approved by the Institutional ethics committee of Kartal Dr. Lutfi Kirdar City Hospital (Ref. No: 2025/010.99/20/22) and was conducted in accordance with the principles of the Declaration of Helsinki.

## Consent

The requirement for informed consent was waived by the institutional ethics committee because of the retrospective design and the use of de‐identified data.

## Conflicts of Interest

The author declares no conflicts of interest.

## Supporting information


**Table S1:** Multivariable Logistic Regression for Clinically Significant Prostate Cancer (csPCa) With PSAD Scaling.
**Table S2:** Ridge (L2‐Penalized) Logistic Regression Sensitivity Analysis for csPCa.

## Data Availability

The data that support the findings of this study are available from the corresponding author upon reasonable request.
