# Supplementary material for: Integrating Prostate‐Specific Antigen Density and Prostate Imaging Reporting and Data System Scores to Optimize Detection of Clinically Significant Prostate Cancer: A Multivariable Risk Model Approach
Source: J Clin Lab Anal. 2026 Apr 4;40(12):e70218. doi: 10.1002/jcla.70218 (PMC13327475; doi:10.1002/jcla.70218)
Supplement: Supplementary file 1 — Table S1: Multivariable Logistic Regression for Clinically Significant Prostate Cancer (csPCa) With PSAD Scaling. Table S2: Ridge (L2‐Penalized) Logistic Regression Sensitivity Analysis for csPCa. [file JCLA-40-e70218-s001.docx]

### Supplementary Table S1. Multivariable Logistic Regression for Clinically Significant Prostate Cancer (csPCa) With PSAD Scaling.

| **Predictor** | **OR (95% CI)** | **p-value** |
| --- | --- | --- |
| PSAD (per 0.1 ng/mL/mL increase) | 1.627 (1.266–2.092) | <0.001 |
| PI-RADS (per 1-point increase) | 2.649 (1.941–3.615) | <0.001 |
| DRE (positive vs negative) | 1.297 (0.625–2.689) | 0.485 |
| Age (per 1 year) | 0.984 (0.946–1.022) | 0.424 |

### *Model specification: PSAD + PI-RADS + DRE + Age*

### *Outcome: csPCa (ISUP ≥ 2) vs non-csPCa (benign + ISUP GG1), n = 375*

*Odds ratios (ORs) and 95% confidence intervals (CIs) were obtained by exponentiating the* corresponding β coefficients and their 95% CIs. p-values were derived from two-sided Wald tests (z = β/SE). Models included an intercept term.

Abbreviations: OR, odds ratio*; CI, confidence interval; PSAD, prostate-specific antigen density; DRE, digital rectal examination; PI-RADS, Prostate Imaging Reporting and Data System; csPCa, clinically significant prostate cancer; ISUP, International Society of Urological Pathology.*

### Supplementary Table S2. Ridge (L2-Penalized) Logistic Regression Sensitivity Analysis for csPCa.

| **A. Standardized Ridge Coefficients (C = 1)** | |
| --- | --- |
| **Predictor** | **Standardized β** |
| PSAD | 0.431 |
| PI-RADS | 0.859 |
| DRE | 0.186 |
| Age | 0.014 |
| Intercept | -1.374 |
| **B. Test-Set Performance (80/20 Stratified Split; random_state = 42)** | |
| **Metric** | **Value** |
| **AUC** | 0.799 |
| **Log-loss** | 0.441 |
| **Brier score** | 0.137 |
| **Penalty parameter (C)** | 1 |

***Outcome:*** *csPCa (ISUP≥2) vs non-csPCa (benign + ISUP GG1), n=375*

*Ridge logistic regression with an L2 penalty was fitted. The parameter C denotes the inverse of the regularization strength. “Standardized β” indicates coefficients estimated on a standardized predictor scale (commonly z-scored continuous variables; binary predictors coded 0/1; PI-RADS treated as an ordinal numeric predictor as above).*

***Abbreviations:*** *AUC, area under the receiver operating characteristic curve; PSAD, prostate-specific antigen density; DRE, digital rectal examination; PI-RADS, Prostate Imaging Reporting and Data System; csPCa, clinically significant prostate cancer; ISUP, International Society of Urological Pathology.*
